# Innovative Approaches to Enhance Preservation Over Invasion in Minimally Invasive Periodontal Surgery: A Narrative Review

**DOI:** 10.7759/cureus.89707

**Published:** 2025-08-09

**Authors:** Shilpi Mangal, Shalini Kaushal, Nand Lal

**Affiliations:** 1 Periodontology, King George's Medical University, Lucknow, IND

**Keywords:** minimally invasive surgery, mist in surgical periodontal therapy, periodontal bone tissue regeneration, periodontal intrabony defects, periodontal microsurgery, periodontal surgery

## Abstract

Minimally invasive surgery (MIS) in periodontology represents a paradigm shift from traditional, trauma-prone surgical procedures to techniques that emphasize tissue preservation, aesthetics, faster recovery, and overall improved patient outcomes. This narrative review delves into the evolution, methodologies, applications, advantages, limitations, and prospects of MIS in periodontal and implant therapy, highlighting key innovations that have refined surgical protocols while enhancing biological and clinical outcomes. The key findings of this narrative review highlight that MIS has significantly advanced periodontal and implant therapy by shifting the focus from extensive tissue resection to preservation, precision, and regeneration. MIS techniques, such as the minimally invasive surgical technique (MIST), modified MIST, vestibular incision subperiosteal tunnel access (VISTA), and tunneling approaches, have demonstrated clear benefits in reducing surgical trauma, enhancing wound stability, and preserving soft tissue architecture, particularly the interdental papillae. These techniques promote faster healing, minimize postoperative complications such as gingival recession, pain, and sensitivity, and offer improved aesthetic outcomes. Furthermore, innovations such as lasers, flapless implant placements, guided sinus augmentation, and soft tissue grafting using allografts have expanded the scope and effectiveness of MIS.

Despite some limitations, such as the need for specialized equipment and operator expertise, MIS offers enhanced patient comfort, reduced morbidity, and excellent regenerative outcomes. The integration of robotic-assisted surgery and 3D-guided navigation indicates a promising future for MIS, aligning surgical precision with patient-centered care and long-term clinical success. The evolution of periodontal surgery toward minimally invasive approaches reflects a transformative shift in clinical thinking from aggressive resection to delicate preservation and regeneration. This review comprehensively underscores that MIS is not merely a surgical philosophy but a revolution in periodontal and implant therapy, aligning the goals of biological preservation, aesthetic perfection, and procedural efficiency. As microsurgical and robotic techniques continue to evolve, the scope and success of MIS will expand, making it the cornerstone of contemporary and future periodontal practices.

## Introduction and background

Periodontitis is a condition characterized by the gradual deterioration of the periodontal tissues that provide structural support for the dentition, which is linked to an unhealthy buildup of plaque biofilm. It represents a chronic inflammation affecting the periodontium [1]. In order to treat advanced cases of periodontitis, conventional flap operations are performed. These surgeries involve the utilization of wide flaps in order to obtain accessibility for the damaged tissues that lie beneath. The primary therapeutic goal of flap surgery is to facilitate direct access to subgingival areas for the mechanical debridement of root surfaces. This enables the thorough removal of plaque biofilm, calculus deposits, and inflamed granulation tissue, which are principal etiological factors in periodontal disease progression. Pathologically deepened periodontal pockets serve as a reservoir for pathogenic bacteria. Flap surgery enables pocket debridement and re-approximation of gingival margins, resulting in a reduction in probing pocket depths. This enhances the patient's ability to maintain adequate oral hygiene and reduces the risk of recurrent periodontal infections.

In cases presenting with intrabony defects, periodontal flap surgery is often combined with regenerative techniques such as bone grafting, guided tissue regeneration (GTR), and the application of growth factors. These adjunctive procedures aim to stimulate the regeneration of alveolar bone, cementum, and periodontal ligament, thereby restoring the structural and functional integrity of the periodontium. Gingival recession and root hypersensitivity may result from some of the traditional techniques, including modified flap operation and modified Widman surgery, as well as resorption of the bone surrounding the tooth, and loss of the interdental papillary gingiva are some of the inevitable sequelae. Any procedure should be simple to execute, result in minimal tissue damage during both the surgery and healing stages, be efficient in terms of time, not impose financial strain on the patient, and ultimately be advantageous for the general population. As conventional techniques utilize a large incision, it can disrupt the existing clot during the healing phase of periodontal flap surgery.

Minimally invasive surgery (MIS) typically involves the use of smaller incisions, minimizes tissue exposure, and limits the mechanical disruption of the existing clot. MIS reduces the need to lift and disturb tissue, thereby minimizing the amount of damage that is done to the blood clot that is present in the wound. This technique helps maintain the crucial blood supply, leading to less shrinkage of the wound after surgery. Minimally invasive surgery enhances tissue healing through multiple interconnected mechanisms. By preserving the vascular supply, it improves blood perfusion and promotes angiogenesis. Its ability to minimize soft tissue trauma leads to reduced inflammation and a more regulated healing cascade. MIS also stabilizes the blood clot, maintaining an intact fibrin matrix that supports granulation tissue formation. Additionally, MIS enhances hard tissue regeneration by promoting improved bone fill and increased clinical attachment level (CAL) gain. Together, these effects create an optimal environment for both soft and hard tissue healing. The principles or the foundation stone of microsurgery are also incorporated into minimally invasive surgery. Daniel (1979) has provided a comprehensive definition [2], that is, "surgical technique that is performed under magnification provided by an operating microscope". Periodontal microsurgery is defined as "refinements in existing basic surgical techniques that are made possible by the use of a surgical microscope and subsequent improved visual acuity" (Shanelec 1992) [3].

Three principles determine the microsurgical approach. The first principle emphasizes the enhancement of surgical capability through the refinement of motor skills. The second principle involves achieving exact primary apposition of the wound edges through passive wound closure. This method allows tissues to come together naturally without excessive tension, thus fostering an environment conducive to optimal healing and minimal scarring. The third advocates for the exclusive use of microsurgical instruments and fine sutures, which are specifically designed to reduce trauma to surrounding tissues during both incision and suturing. Collectively, these principles form the cornerstone of microsurgical techniques in periodontics, enhancing both the precision of procedures and the overall patient experience [4].

The need for a review of minimally invasive periodontal surgery arises from the growing emphasis on preservation-oriented, patient-centered, and evidence-based surgical practices in periodontology. Traditional periodontal surgeries, while effective, often involve extensive flap elevation, greater soft tissue trauma, and longer recovery times, which can lead to increased patient discomfort, gingival recession, and compromised aesthetics. In contrast, minimally invasive surgery techniques represent a refined surgical modality characterized by the use of smaller incisions, enhanced visual magnification, and micro-surgical instrumentation. These approaches emphasize the preservation of soft and hard tissues, minimize surgical trauma and postoperative morbidity, and are associated with accelerated wound healing and improved patient recovery outcomes.

Despite the documented advantages, many clinicians remain unfamiliar or undertrained in these newer techniques. Many clinicians develop confidence and routine around established methods, and may be reluctant to shift to unfamiliar techniques that could require: New instruments or technology, different healing protocols, and a learning curve. A comprehensive review is therefore essential to synthesize and critically evaluate these diverse methodologies, providing clarity on their effectiveness, scope of application, and comparative benefits over conventional approaches.

This review article offers a novel perspective by specifically focusing on minimally invasive surgical approaches such as minimally invasive surgical technique (MIST), single flap approach (SFA), and examines emerging techniques like entire papilla preservation technique (EPPT), non-incised papillae surgical approach (NIPSA), apically incised coronally advanced surgical technique (AICAST), vestibular incision subperiosteal tunnel access (VISTA), and non-incisional technique (NIT). These techniques aim not only for clinical regeneration but also prioritize preservation of soft tissue aesthetics and vascular integrity, which are crucial in anterior esthetic zones, drawing attention to their potential for improved patient-centered care.

## Review

Methods

Information Sources and Search

The following scientific sources were searched from 1970 to 2024 via PubMed MEDLINE, Scopus, and Web of Science. A search strategy was finalized utilizing Medical Subject Headings (MeSH) terms and free text keywords using Boolean operators (AND/OR). In addition, a manual search of the reference list of the potential studies was performed to retrieve additional articles. Duplicate articles were removed. The following MeSH terms were used: ("periodontal surgery"(MeSH) OR "minimally invasive surgical procedures"(MeSH) OR "MIST" OR "tunneling technique" OR "VISTA") AND ("bone regeneration"(MeSH) OR "root coverage" OR "soft tissue grafting") AND ("clinical trial" OR "case series" OR "comparative study"). All the authors unanimously carried out a thorough search.

Eligibility Criteria

Inclusion Criteria: Randomized trials and non-randomized prospective studies, case series, narrative reviews, meta-analyses, and all types of human studies (studies) on the young and adult population >18 years of age focused on clinical applications or innovations in minimally invasive periodontal techniques, describing novel surgical techniques (e.g., MIST, VISTA, NIPSA, Whale's Tail), reports showing clinical outcomes (e.g., CAL gain, gingival recession), studies emphasizing tissue preservation, aesthetic outcomes, and reduced morbidity included.

Exclusion Criteria: All studies conducted in vitro, animal studies, mini-reviews, and conference proceedings, not focused on surgical techniques (e.g., pharmacological studies), studies with unclear methodology, or insufficient clinical data, articles published in languages other than English were not considered.

To ensure that the inclusion of studies satisfies the established criteria, the selection procedure consisted of evaluating article titles, abstracts, and full texts. A summary of the search strategy and hits across databases is presented in Table 1.

**Table 1:** Summary of search strategy and database hits

Database	Search strategy	Hits retrieved
MEDLINE (PubMed)	("Periodontal Surgery"[MeSH] OR "Minimally Invasive Surgical Procedures"[MeSH] OR "MIST" OR "tunneling technique" OR "VISTA") AND ("Bone Regeneration"[MeSH] OR "Root Coverage" OR "Soft Tissue Grafting") AND ("clinical trial" OR "case series" OR "comparative study"	392
Web of Science	("Periodontal Surgery"[MeSH] OR "Minimally Invasive Surgical Procedures"[MeSH] OR "MIST" OR "tunneling technique" OR "VISTA") AND ("Bone Regeneration"[MeSH] OR "Root Coverage" OR "Soft Tissue Grafting") AND ("clinical trial" OR "case series" OR "comparative study"	150
Scopus	("Periodontal Surgery"[MeSH] OR "Minimally Invasive Surgical Procedures"[MeSH] OR "MIST" OR "tunneling technique" OR "VISTA") AND ("Bone Regeneration"[MeSH] OR "Root Coverage" OR "Soft Tissue Grafting") AND ("clinical trial" OR "case series" OR "comparative study"	200
TOTAL		742

Study Selection Process

**Figure 1:**
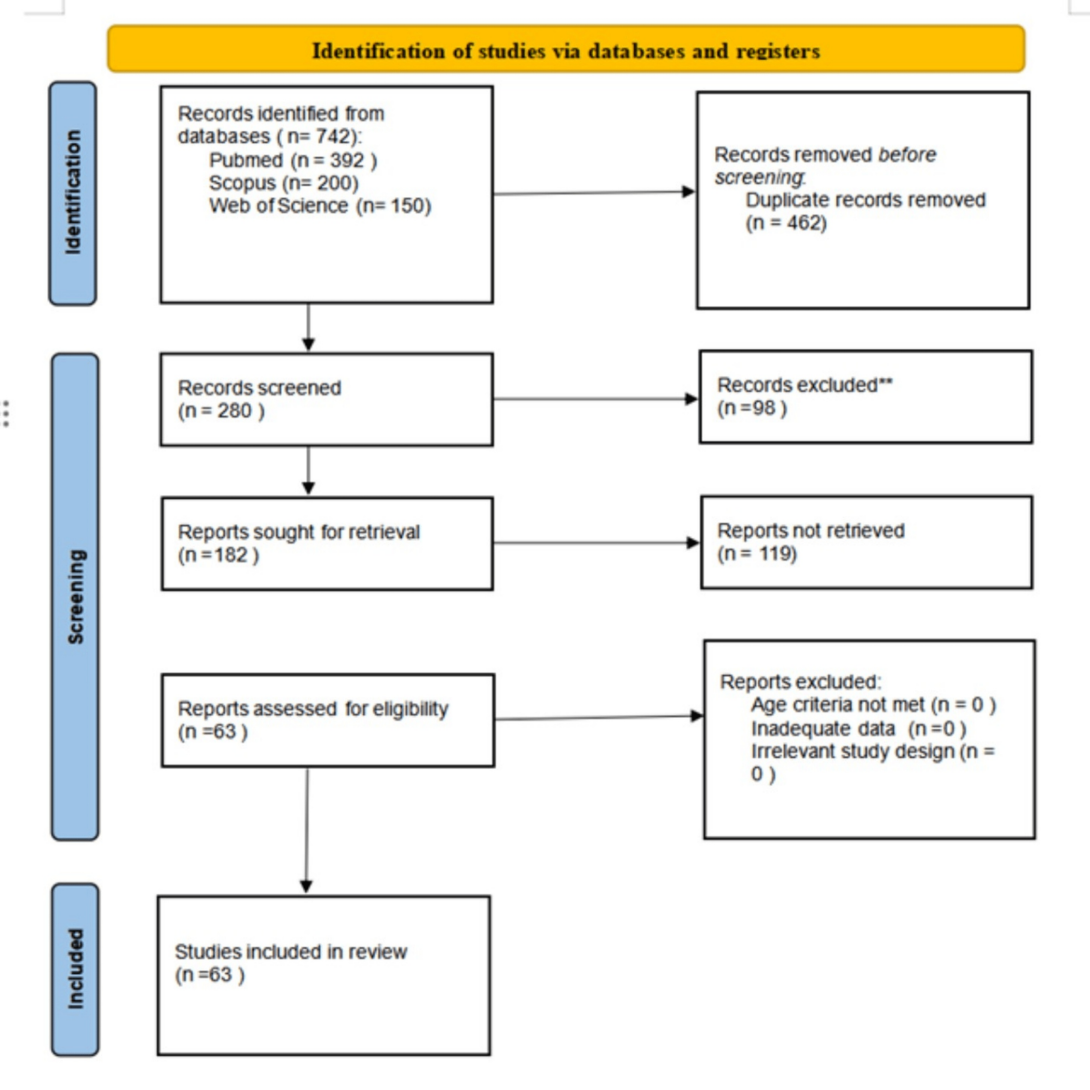
PRISMA 2020 flow diagram illustrating the study selection process for inclusion in the review PRISMA - Preferred Reporting Items for Systematic reviews and Meta-Analyses

A total of 742 articles were initially retrieved from three databases: MEDLINE (PubMed) (n=392), Scopus (n=200), and Web of Science (n=150), based on a comprehensive search strategy targeting studies on clinical applications and innovations in minimally invasive periodontal techniques. After the removal of 462 duplicate records, 280 unique articles were subjected to title and abstract screening by two independent reviewers. Based on the predefined eligibility criteria, which included randomized and non-randomized human studies, case series, and reviews involving adult populations (>18 years) with a focus on surgical techniques such as MIST, VISTA, NIPSA, and Whale's Tail, 98 studies were excluded. The full texts of 182 potentially relevant articles were then assessed for eligibility. Articles were excluded if they were in vitro or animal studies, pharmacological in nature, had insufficient clinical data, or were published in languages other than English. Disagreements during the selection process were resolved by consensus with a third reviewer. Ultimately, 63 studies met all inclusion criteria and were included in the final qualitative synthesis. The entire study selection process is outlined in the Preferred Reporting Items for Systematic reviews and Meta-Analyses (PRISMA) 2020 flow diagram (Figure 1).

Historical perspective

Serafin [5] defined microsurgery in 1980 as "a methodology- a modification and a refinement of recent surgical procedures". Carl Nylen, widely acknowledged to be the "father of microsurgery", performed ear surgery in 1921 using a crude binocular microscope. It was in the year 1694 that the first compound lens microscope was created by Anton van Leeuwenhoek, a merchant from Amsterdam. Saemisch, a German ophthalmologist, was the first person to introduce basic binocular loupes for use in ophthalmic surgery in the year 1876. Barraquer [6] implemented the microscope in corneal surgeries during the 1950s. In 1978, the microscope was initially introduced to dentistry by Apotheker and Jako. Wickham and Fitzpatrick [7] came up with the term "minimally invasive surgery" in the year 1990. This term was used to describe surgical procedures that involve the utilization of smaller incisions.

The notion of minimally invasive surgery (MIS) was further refined by Hunter and Sackier (1993) [8]. They also provided a description regarding the surgical approach, "the capacity to miniaturize our eyes and extend our hands to perform microscopic and macroscopic operations in locations that could previously only be accessed through large incisions". Shanelec and Tibbetts [9] conducted a continuing development workshop on periodontal microsurgery, which was delivered during the yearly conference of the American Academy of Periodontology in 1996. In 1995, Harrel and Ress [10] proposed MIS intending to decrease the embarrassment associated with the supply of blood through the production of "minimal wounds, minimal flap reflection, and gentle handling of hard and soft tissue" [11].

Preserving the original gingival structure before surgery and restoring the gum papilla to its original location or slightly above it are two key factors in reducing the risk of gingival resorption, a distinguishing characteristic of this procedure [12]. In 2001, Belcher presented a condensed summary of the potential applications and advantages of utilizing a surgical microscope during periodontal therapy [12]. Microsurgical instruments, surgical telescopes (loupes), and operating microscopes all contributed to an improvement in the prognosis of surgery (Cortellini et al. 2001, 2005) [13,14]. Cortellini, along with Tonetti, initially introduced the minimally invasive surgical technique (MIST) in 2007 [15,16]. With the intention of prioritizing the stability of wounds and blood clots, MIST was created, as well as the closure of wounds to protect against blood clotting. Through the implementation of modified minimally invasive surgical technique (M-MIST, Cortellini, along with Tonetti, 2009), Cortellini and Tonetti advanced it even further by incorporating the concept of providing space for regeneration. As an alternative, the simplified papilla preservation flap (SPPF) is utilized to obtain entry to the defect-related interdental papilla in the MIST [17] approach within narrow interdental spaces. Alternatively, the modified papilla preservation method (MPPT) [18,19] is used in interdental spaces that are substantial.

Rationale of minimally invasive surgery

The rationale behind minimally invasive surgery (MIS) in periodontology is deeply rooted in the goal of optimizing clinical outcomes while minimizing patient burden. One of the primary objectives is the minimization of surgical trauma, achieved through smaller incisions and less tissue manipulation, which in turn fosters a more favorable healing environment. Equally important is the augmentation of flap or wound stabilization, as stable surgical sites are crucial for clot integrity and tissue regeneration. MIS also focuses on enhancing the efficiency of wound closure, promoting direct sealing of the wound edges to facilitate rapid and predictable healing. Another significant advantage is the reduction in operative time, which not only increases clinical efficiency but also contributes to greater patient comfort by minimizing the physical and psychological strain associated with prolonged procedures. Furthermore, MIS techniques are associated with lower intraoperative and postoperative morbidity, reducing complications such as pain, swelling, and tissue trauma. Finally, these methods are instrumental in the prevention of postoperative gingival recession, preserving the natural contour and aesthetics of the gingiva [20].

Comparison of minimally invasive periodontal surgery and conventional periodontal surgery is shown in Table 2.

**Table 2:** Comparison of conventional and minimally invasive periodontal surgery

Comparison criteria	Conventional periodontal surgery	Minimally invasive periodontal surgery
Incisions	Flaps that have been designed to extend at least one tooth on each side of the periodontal defect, and they are made wide.	The procedure is limited to the facial or lingual aspect and is performed with smaller incisions, affecting only the immediate vicinity of the periodontal defect and not the healthy teeth.
Flap reflection	The procedure entails the extensive lifting of soft tissue flaps, which involves reflecting the tissue from the underlying bone to fully expose all of the supporting bone. Additionally, vertical releasing incisions at the border of the flap could potentially be employed.	Facilitates reduced tissue manipulation, which reduces the overall trauma to surgical sites and enables faster healing. The periosteum is preserved, and the tissue is sharply dissected to the bone's level. That tissue is not elevated from the remaining bone.
Surgical closure	Multiple interrupted sutures.	A single mattress suture per surgical site.
Results	The primary objective is bone regeneration. The treatment of periodontal defects typically results in 2-4 mm of gingival recession due to the excessive apical displacement of the gingival margin.	The objective is to promote bone regeneration, with a minimum of 0.05 mm of gingival recession that is clinically undetectable following the operation.
	Enhanced patient morbidities, including compromised aesthetic outcomes, interproximal food impaction, and thermal sensitivity.	Patient morbidity and chair time were reduced to a minimum.
Armamentarium	Loupes, microscopes, and microsurgical instruments are unnecessary.	Utilization of loupes, microscopes, and other instruments used in microsurgery.

Indications

One primary indication is the presence of interproximal bone defects that are isolated and confined to the interproximal site, making them ideal for localized treatment without extensive flap elevation. Additionally, edentulous areas that are adjacent to periodontal defects present suitable conditions for MIS, as these sites can be managed effectively with minimal disruption to surrounding tissues. The technique is also beneficial in cases involving periodontal defects that extend from the interproximal area to the buccal or lingual regions, allowing for targeted access and regeneration without the need for wide exposure. Moreover, multiple distinct defect sites within a single quadrant can be addressed efficiently through minimally invasive approaches, reducing overall surgical trauma and improving patient comfort during multi-site treatment. These indications highlight the versatility and precision of MIS in managing complex periodontal conditions while preserving anatomical and functional integrity [21].

Contraindications

Minimally invasive periodontal surgery has several benefits, but also has contraindications that must be considered during case selection. One significant limitation is the presence of generalized horizontal bone loss, where the uniform nature of the defect across multiple sites makes it challenging to localize treatment through small, precise incisions. In such cases, broader surgical access is often required to manage and regenerate the affected areas adequately. Additionally, interconnected vertical defects and complex multi-walled bony structures pose difficulties for minimally invasive approaches due to limited visibility and restricted instrument access. These anatomical complexities may compromise the ability to achieve effective debridement, graft placement, and flap management, thereby reducing the predictability of treatment outcomes. Therefore, cases exhibiting these contraindications are better suited for more conventional surgical techniques that allow comprehensive access and control [21].

Instruments required for minimally invasive surgery are shown in Table 3.

**Table 3:** Instruments required for minimally invasive surgery

Instruments	Examples
Knives	Blade breaker knife, crescent knife, mini crescent knives, spoon knife, lamellar knife
Microsurgical blades	Ophthalmic blades no. 15,12, 390 c
Microsurgical periosteal elevators	Prichard periosteal (PPSCHLEE), Hourigan periosteal (PH2MBHKD)
Microsurgical tissue forceps	Microsurgical anatomic tissue pliers TPASTMBH. Microtissue forceps 180
Microsurgical needle holder	Microneedle holder Schlee (NHSLSCHLEE)
Microscissors	Micro-vannas tissue scissors, Goldman-Fox scissors, Ligature scissor FD252R
Microsurgical needles	Reverse cutting needles with precision tips
Microsutures	6-0 to 10-0

General factors to consider when performing minimally invasive surgery

Foremost, all incisions are designed with the primary goal of preserving soft tissue integrity, avoiding unnecessary trauma to surrounding structures. To achieve this, continuous incisions are typically avoided, and instead, separate, strategically placed incisions are employed to minimize disruption. Vertical releasing incisions are deliberately omitted to maintain vascular supply and aesthetic contours, especially in anterior regions. In aesthetically sensitive areas, such as the anterior maxilla, surgical access is achieved through the palatal papilla, allowing soft tissue to completely cover the graft or membrane and support periodontal regeneration [21]. Tissue reflection is carefully performed using sharp, blunt, or combined dissection techniques, depending on the surgical requirement and the nature of the tissues involved. Proper visualization is crucial, necessitating the use of a light source and magnification tools, such as 3.5× loupes or surgical microscopes, to enhance precision. However, the limited flap reflection in MIS can make root surface debridement more technically demanding. To address this, clinicians utilize vertical positioning of curettes and maintain the shank parallel to the root surface, supplemented by ultrasonic scalers to disrupt granulation tissue effectively [21]. The placement of bone graft materials is facilitated with high accuracy using instruments like a plastic plunger gun, ensuring proper adaptation within the defect. For wound closure, vertical mattress sutures are commonly used to approximate tissues between adjacent teeth, with 6-0 resorbable sutures being the preferred choice to minimize tension and support primary closure [22].

Advantages of MIST in periodontal regeneration

One of its primary benefits is the reduction in the postoperative healing phase, allowing for quicker tissue recovery and earlier functional restoration. MIST also minimizes common postoperative complications such as edema, pain, and root sensitivity, which contributes to a more comfortable recovery experience. Additionally, the technique is designed to reduce or eliminate scarring by minimizing both flap reflection and tissue manipulation, particularly in the delicate papillary regions, thus achieving superior aesthetic outcomes. This meticulous handling of tissues contributes to enhanced height and contour of the papillary soft tissue, preserving the natural appearance of the gingiva. Importantly, MIST significantly limits or prevents postoperative gingival recession, a frequent concern in traditional procedures, thereby maintaining the integrity of the periodontal architecture. These clinical advantages are further reflected in a notably improved patient receptiveness and satisfaction, as the approach aligns with patient expectations for minimally traumatic, effective, and cosmetically favorable treatment options [22].

Disadvantages of MIST

One of the primary drawbacks is its technique-sensitive nature, largely due to the limited accessibility afforded by the small surgical windows. This restriction demands a high level of precision and control, making the procedure highly dependent on the operator's skill and experience. Moreover, the successful implementation of MIST requires specialized instruments, including microsurgical tools, magnification loupes, and operating microscopes, which may not be readily available in all clinical settings. These requirements can lead to increased procedural costs, potentially limiting accessibility for both clinicians and patients. In addition, the complexity of the technique may result in longer operative times, especially for practitioners who are not yet proficient in minimally invasive protocols. Anatomical challenges, such as difficulty in accessing palatal defects through the small buccal window, can further complicate treatment and restrict its application. Finally, it is important to note that MIST is not universally suitable for all periodontal defects, particularly those requiring broader exposure or extensive regeneration. These limitations highlight the importance of careful case selection, advanced training, and proper instrumentation when considering MIST as a treatment modality [22].

MIS for flap surgery

**Figure 2:**
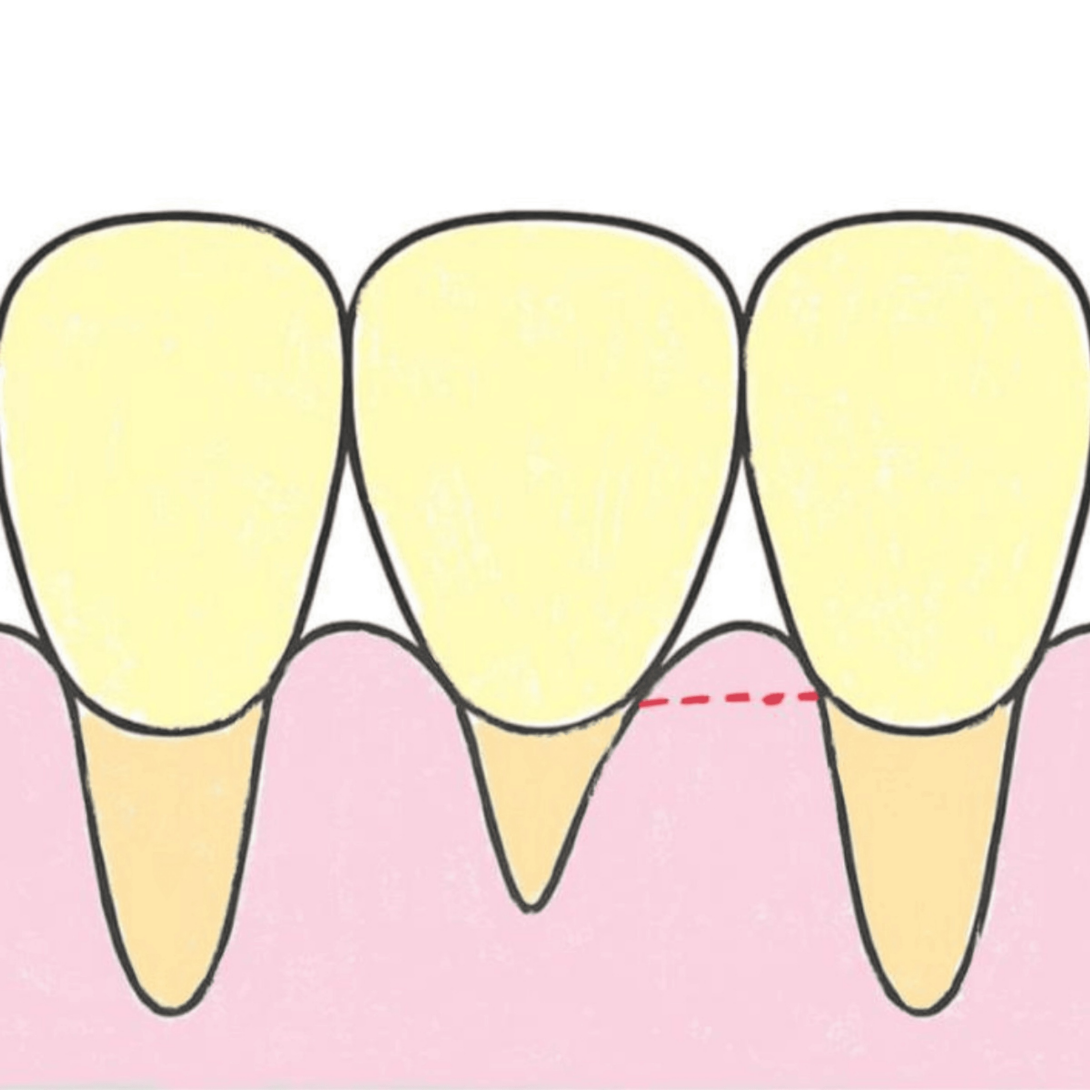
Modified papilla preservation technique Original figure by author

**Figure 3:**
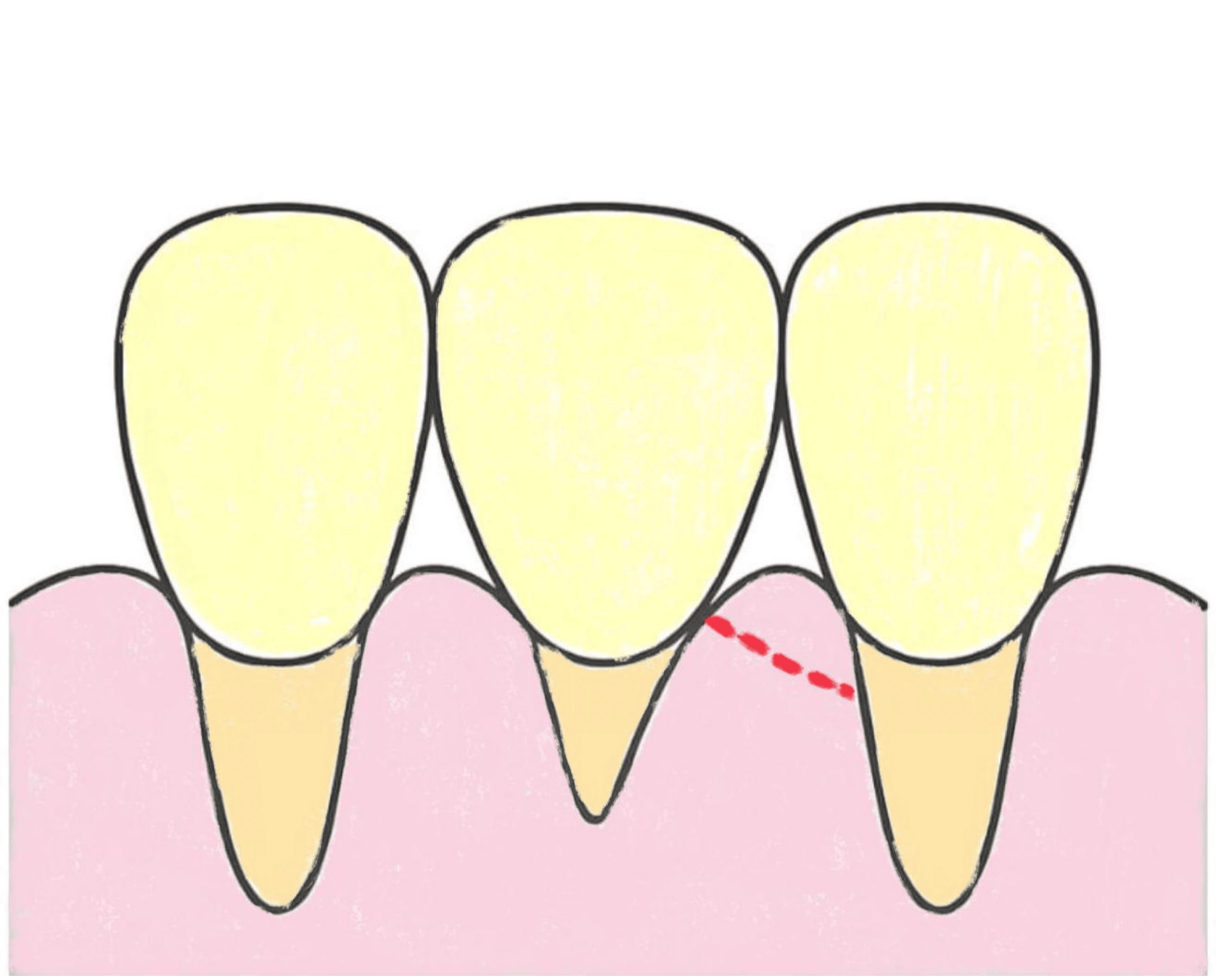
Simplified papilla preservation flap Original figure by author

The modified papilla preservation technique (MPPT) constitutes a horizontal incision that takes place on the buccal portion of a papilla (Figure 2), whereas the simplified papilla preservation flap (SPPF) is a diagonal incision that is traced as precisely as possible to the buccal portion of the papilla colon (Figure 3); both incisions are performed on the papilla colon [23].

Single flap approach (SFA) is a surgical procedure that involves creating a flap in the buccal region without making any vertical releasing incisions. Sulcular incisions are made along the gingival margin of the teeth within the surgical area. The mesiodistal extension of the flap is restricted to maintain accessibility for the installation of bone biomaterial or membrane, as well as for the surgical removal of defects [24].

**Figure 4:**
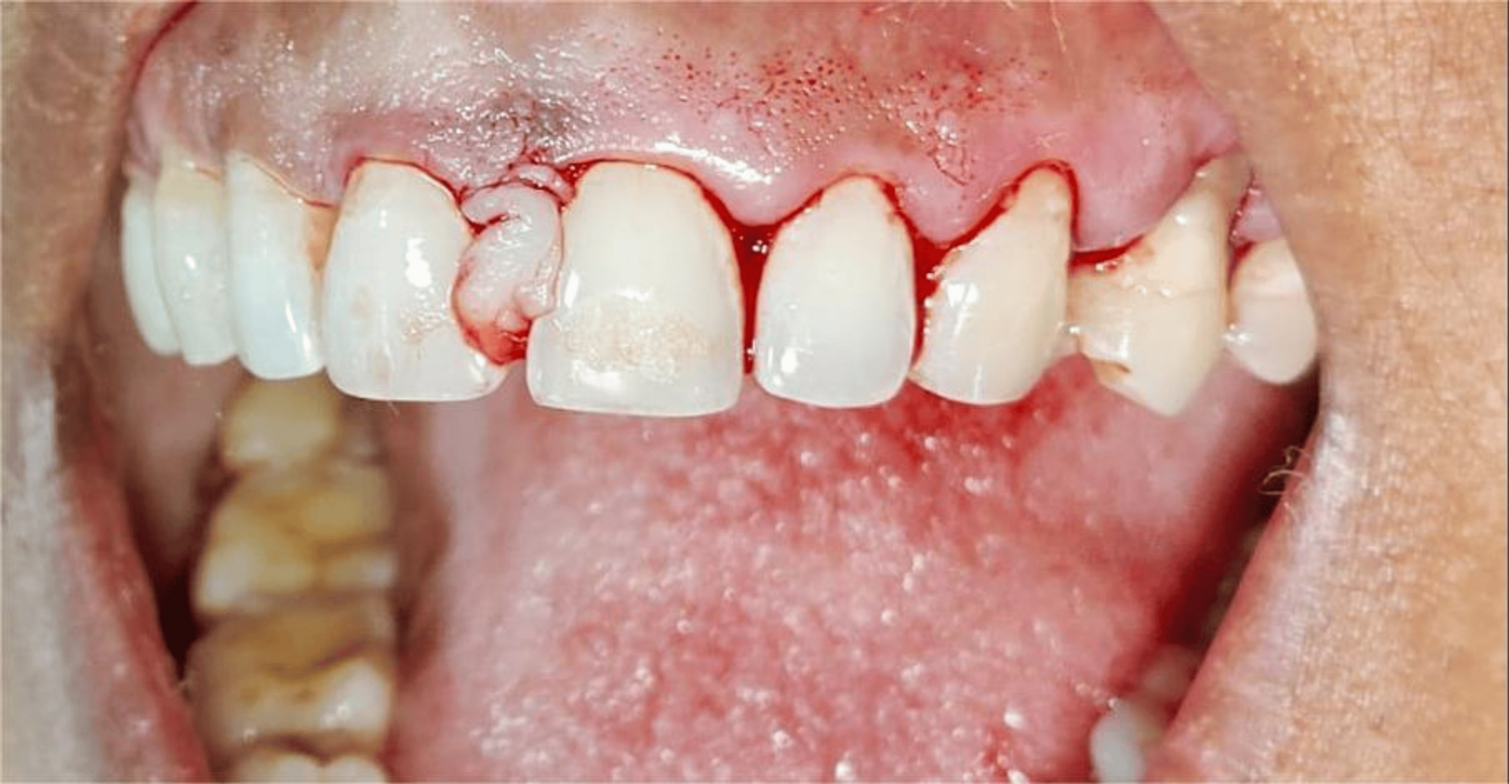
Entire papilla preservation Original figure by author

Entire papilla preservation flap (EPP)- Buccal intracrevicular and single short vertical releasing incision, followed by interdental tunnel preparation below the papilla to access the defect [25] (Figure 4).

Non-incised papilla surgical approach (NIPSA)

The non-incised papillae surgical approach (NIPSA) was originally designed and introduced by Rodriguez et al. Buccal horizontal incision apical to the periodontal defect, followed by raising the flap coronally, allowing surgical access to the defect without disrupting marginal tissues [26].

Whale's technique

**Figure 5:**
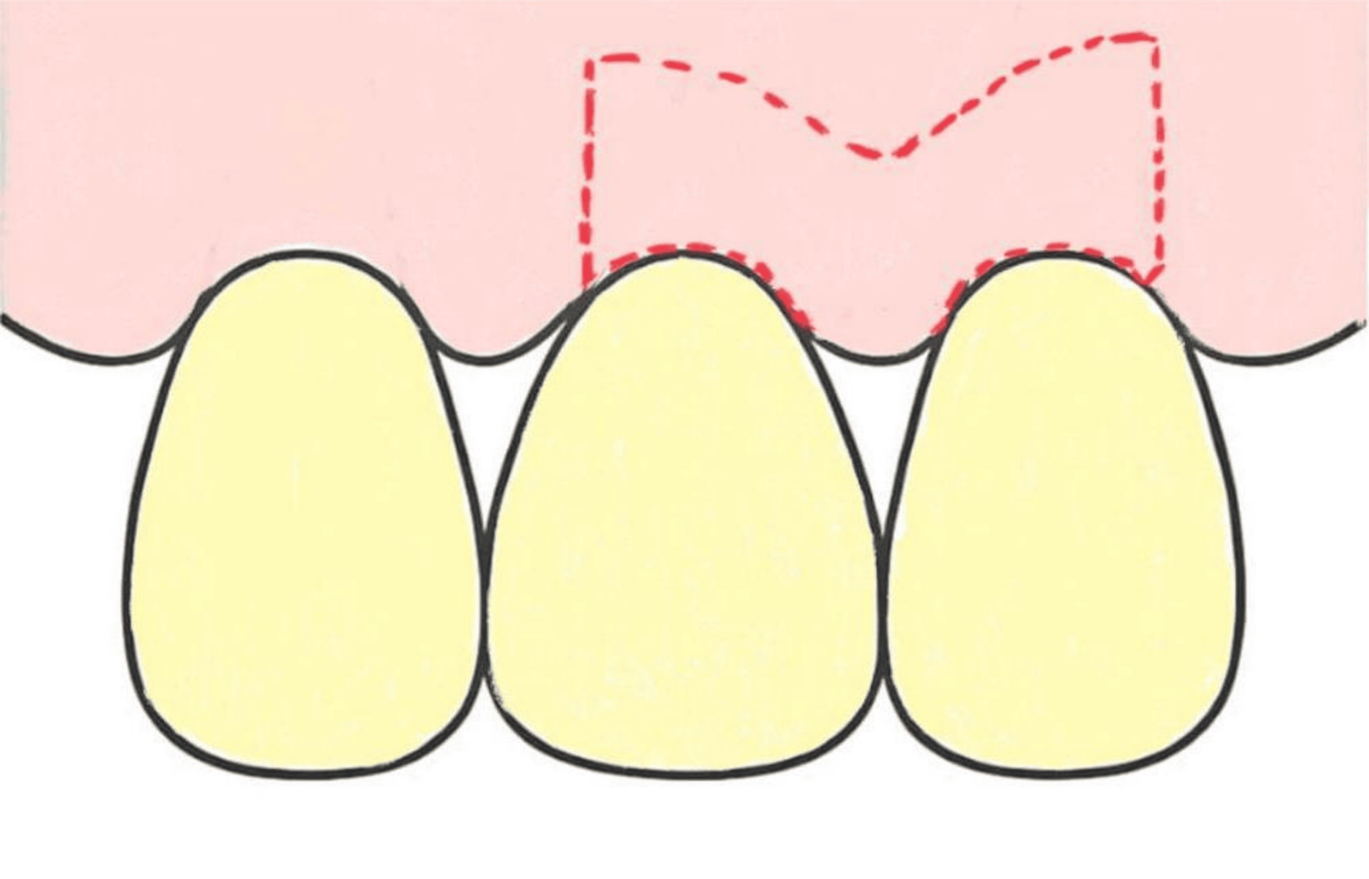
Whale's technique Original figure by author

Introduced by Bianchi & Bassetti in 2009, this treatment is used for addressing extensive intrabony defects in the aesthetic zone. By maintaining interdental tissue over grafting material, this technique involved elevating a large flap across the buccal area to the palatal portion of the mouth (Figure 5). This approach helps to maintain the tissue between teeth and allows for the restoration of a functional attachment with pleasing aesthetic outcomes [27].

Modified Whale's tail

This procedure is employed to attain complete closure and stimulate the regrowth of a deficiency in the bone between teeth. Two incisions in the shape of crescents were constructed on both sides of the frenum; no sutures are necessary at the level of the papillae. This eliminates the potential for suture materials to undergo a "wicking effect" [28].

MIS for root coverage

**Figure 6:**
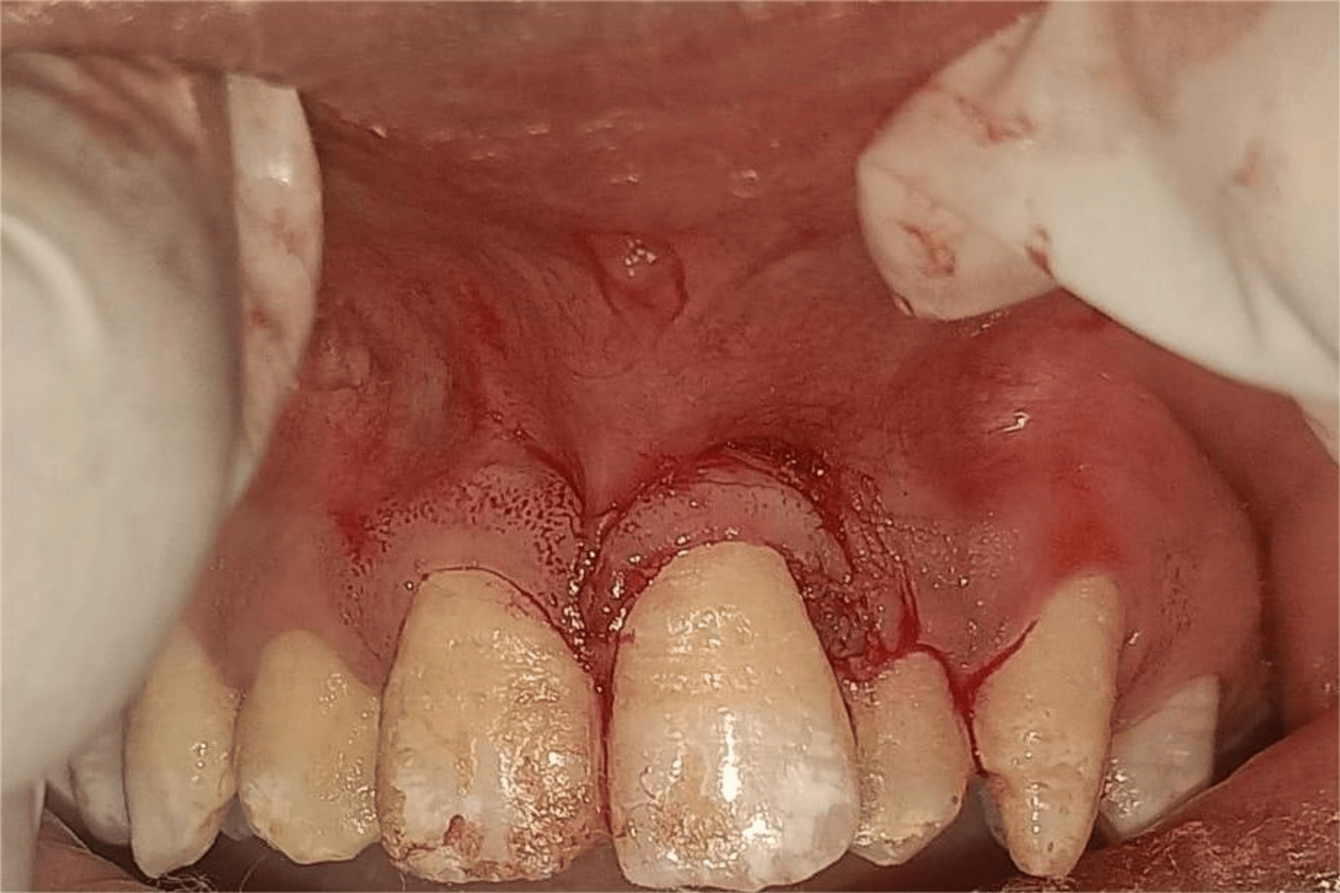
Semilunar technique Original figure by author

At present, there is an increasing inclination towards minimally invasive surgical methods that have the potential to produce satisfactory aesthetic results while minimizing patient morbidity. Semilunar technique, which was first reported by Tarnow in 1986 [29], involves creating a scalloped incision within the attached gingiva in addition to the alveolar mucosa (Figure 6).

The tunneling technique was first presented by Allen in the year 1994, and it was then adopted by Zabalegui et al in the year 1999, respectively, [30,31], resulting in the formation of a "tunnel" or "multi-envelope recipient bed" that includes all of the teeth that have been impacted by recession.

Modified Tunnelling: Mahn's 2001 description marked the introduction of the modified tunneling technique, which requires the insertion at each end of the tunnel flap, two vertical releasing incisions (VRIs). These incisions aid in the placement of the graft and the advancement of the coronal flap [32].

**Figure 7:**
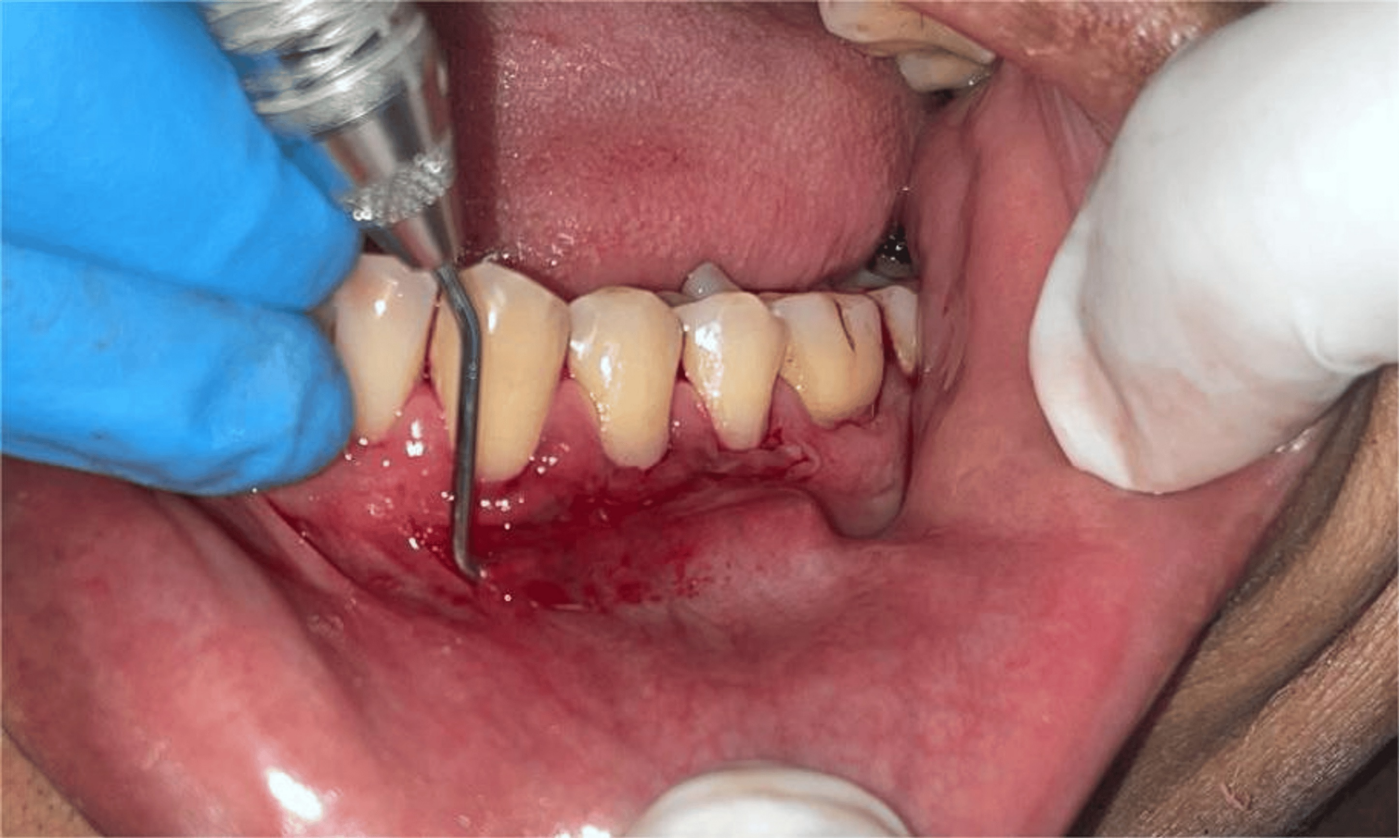
Vestibular incision subperiosteal tunnel access Original figure by author

Vestibular Incision Subperiosteal Tunnel Access (VISTA) Technique: Zadeh originally explained the VISTA method in 2011 [33]. The procedure commences by making a vertical access incision in nonkeratinized tissue through the vestibular full thickness (Figure 7).

**Figure 8:**
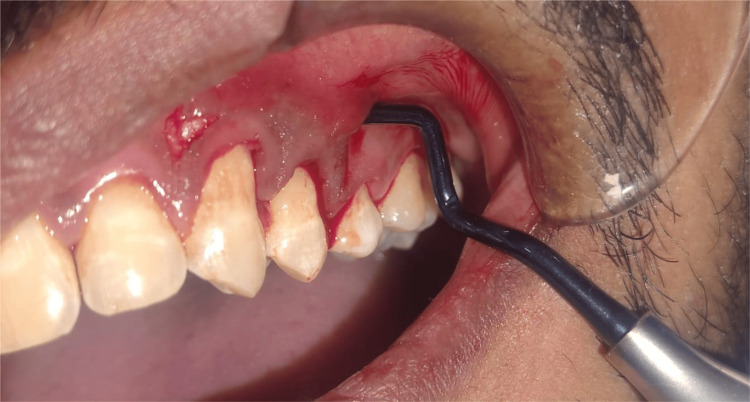
Modified vestibular incision subperiosteal tunnel access Original figure by author

Modified VISTA technique was initially described by Lee et al. in 2015 [34], the technique utilises a supra-periosteal flap design as opposed to the original sub-periosteal approach (Figure 8).

Lingual Incision Subperiosteal Tunnel Access: Intrasulcular incisions were made around the affected tooth using microsurgical blades and specially designed tunnelling knives, following a mucosal lingual access incision. The graft was subsequently secured using a double-crossed suture technique devised by Otto Zuhr, either at the cementoenamel junction or 1 mm below it [35].

The mixed thickness tunnel technique (MiTT) is a direct and efficient method for performing minimally invasive surgery. It is a viable choice, with a high success rate, in addition to maintaining an appealing look, predictability is essential [36].

**Figure 9:**
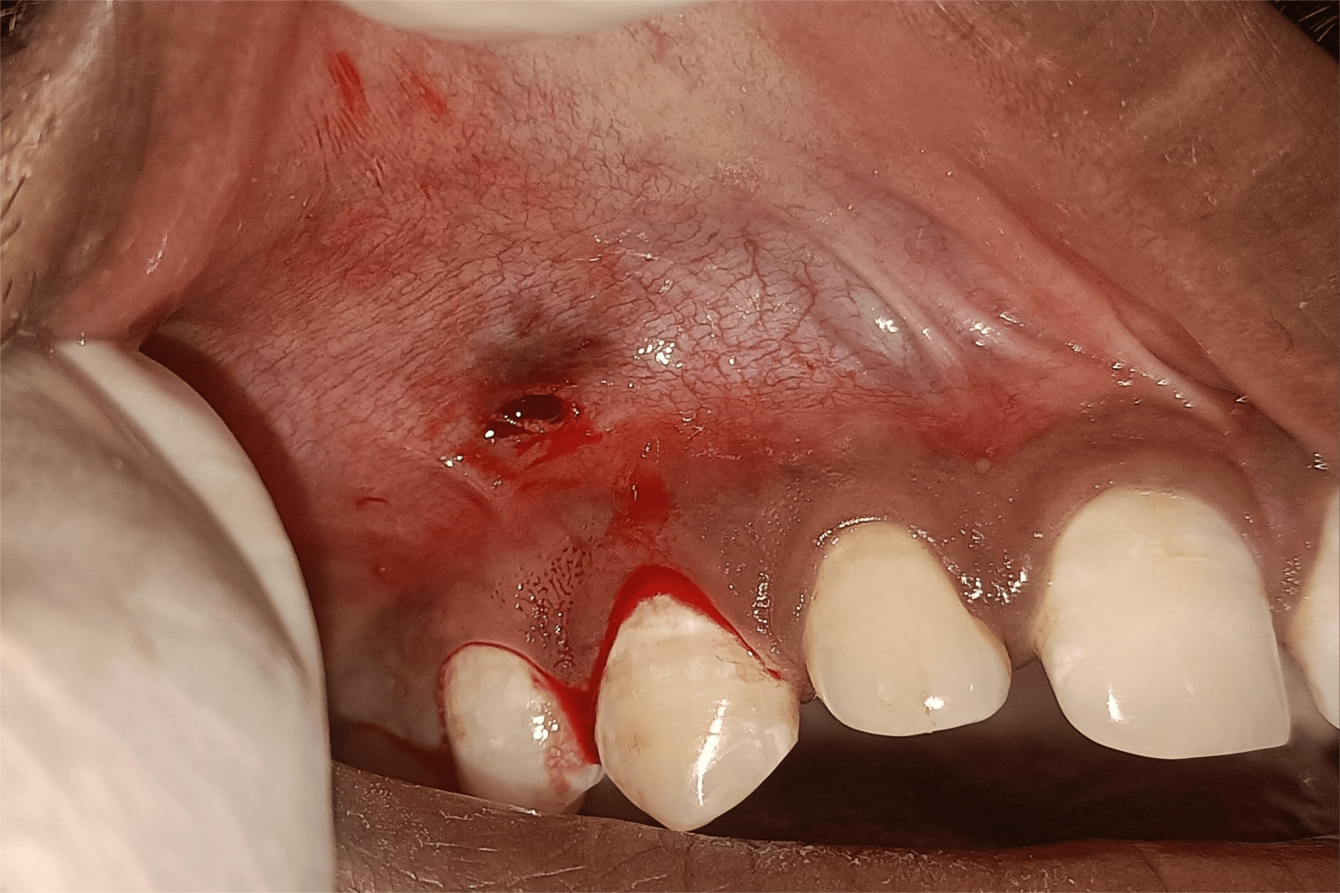
Pinhole technique Original figure by author

Pinhole technique, which was first presented by Chao in 2012, is a minimally invasive technique used for recession coverage in CAF procedures [37] (Figure 9).

MIS for soft tissue grafting

The elimination of the palatal donor site is identified as an important feature of minimally invasive soft tissue transplantation. The tunnel approach is a less intrusive way of preparing a site that is appropriate for both using a person's own tissue (autologous) and using tissue from a different person (allogeneic). One notable benefit of using allografts is the ability to treat multiple teeth in a single visit without worrying about the availability of palatal tissue [38].

MIS for ridge augmentation

Vascularized Interpositional Periosteal-Connective Tissue Graft (VIP-CT)

Tunnel concept for ridge augmentation can also be utilized to address alveolar ridge deficiencies by utilizing rotating autogenous palatal connective tissue grafts. When referring to the "vascularized interpositional periosteal-connective tissue graft" (VIP-CT), Sclar uses the phrase "rotated palatal pedicle graft" to describe the procedure [39,40].

Split Crest: Immediate Expansion Rim Technique

**Figure 10:**
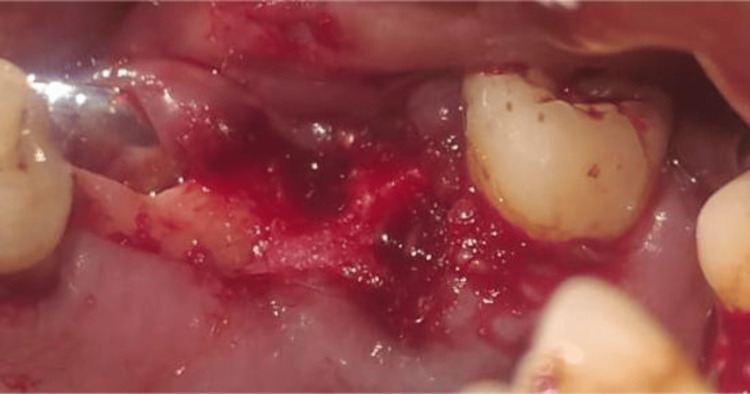
Split ridge technique Original figure by author

The denser palatal cortex prevents the expansion from going as far as it might; thus, a partial osteotomy of the vertical ridge must be planned (Figure 10). The ridge's width increases, and the lateral displacement occurs as a result of the cortical bone's easy expansion [41]. In a study by Triplett et al [42], it was discovered that the enlarged area had a substantial amount of osteogenic activity.

Bone core technique

It is necessary to completely put the implant within the boundary of the bone, which is a critical criterion in this technique. The alveolar crest is exposed following a midcrestal incision that reflects a mucoperiosteal flap. The preparation of the implant bed commenced with the use of a trephine bur, which was also used to harvest a bone core graft that was approximately 10 mm in length. A core removal instrument is then employed to extract the core. Using the original implant system burs, the preparation of the implant bed was carried out until it reached its ultimate length and breadth without the use of irrigation, while low-speed drilling (80 rpm) was employed to collect additional bone chips. The implant is then placed into the bony outlines, and bone chips are used to hide the threads that are exposed [43].

Lasers in minimally invasive periodontal surgeries

Two dentists in California developed the laser-assisted new attachment procedure (LANAP) in the 1990s, which employs an Nd: YAG laser. LANAP is a minimally invasive surgical procedure. This is supported by the systematic review conducted by Kao et al. [44], in the American Academy of Periodontology Workshop, among other things, a recent assessment according to Aoki et al. [45].

**Figure 11:**
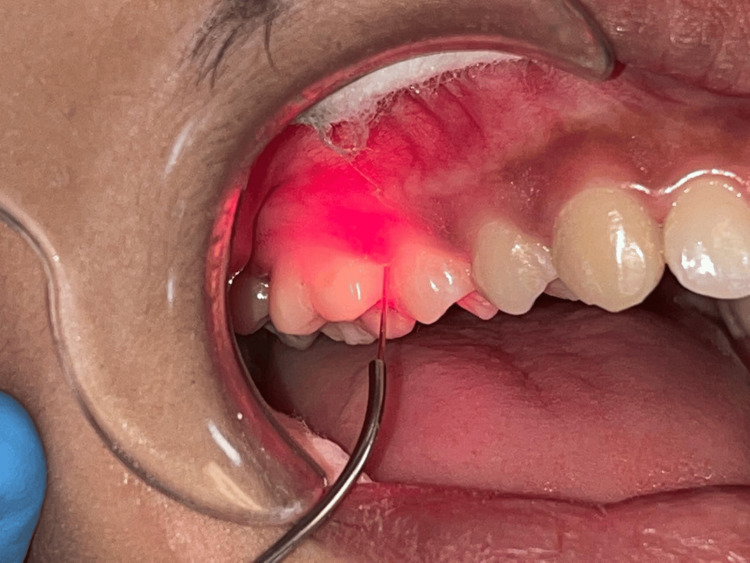
Low level laser therapy Original figure by author

The term "photobiomodulation" refers to a specific kind of laser therapy that includes the delivery of light energy, also known as photons, to a specific tissue (Figure 11). The term "low-level laser therapy" is another name for this particular use of laser therapy. Additionally, it has been demonstrated that the effect of photobiomodulation can significantly speed up the process of tissue regeneration and restoration in nonsurgical treatments [46-48].

MIS in sinus floor elevation

Several authors have proposed modifications to traditional techniques, leading to the establishment of 'Minimally invasive Techniques' as a permanent solution (Kfir et al. in 2013) [49]. The innovative implant technology (IIT) Sinu Lift System is one of the procedures used for minimally invasive indirect sinus lift. Devices that were developed as a result of the search for an alternative way for sinus augmentation. Hatch Reamer, Sinu-Lift System, Sinus Master, Sinus Crestal Approach (SCA) kit, Dentium Advanced Sinus Kit, sinus lateral approach kit, Dr. Cosci Drill, and Sinus Lift Drill are among the numerous minimally invasive sinus lift devices that are currently available on the market [50].

Balloon elevation technique

The antral membrane balloon elevating method is a further procedure that is considered to be minimally invasive, which can be utilized to elevate the sinus membrane from its previous position. By utilizing an inflating balloon, the sinus membrane is brought to a higher position [50].

Minimally invasive transcrestal sinus augmentation approach using calcium phosphosilicate putty

Kher et al. (2014) explored an additional innovative approach that assessed a simplified transalveolar sinus elevation approach that utilized calcium phosphosilicate (CPS) putty to elevate the sinus membrane, which is hydraulic. This treatment was minimally invasive [51].

A modification of Summers' technique is employed to perform transcrestal sinus floor elevation in this particular method. A cautious injection of CPS putty measuring half a centimeter is carried out through osteotomy. The strain of the putty's hydrostatic force causes the sinus floor to be elevated. When CPS putty is added in increments, it is possible to notice intraoperative radiographs that show a sufficient elevation of the Schneiderian membrane following the procedure [51].

Minimally invasive technique sinus augmentation (MITSA)

Has been utilized in conjunction with Osseo densification (OD), a pioneering biomechanical method for preparing osteotomies without excavation, developed by Huwais in 2013 [52]. Osseodensification is performed by utilising specialised burs called Densah™ burs, which aid in compacting bone while creating an osteotomy [53].

Computer-aided design/computer-aided manufacturing (CAD/CAM) approach

Pozzi and Moy proposed an innovative sinus elevation technique that integrates a guided surgical approach and computer-assisted planning. This is accomplished by employing expander-condensing osteotomes in conjunction with a surgical template that is computer-aided design/computer-aided manufacturing [54].

MIS for dental implants

Although minimal invasiveness in implantology encompasses a variety of concepts, it is primarily associated with flapless procedures when it pertains to implant placement. The placement of flapless implants has been shown to lower the amount of surgical trauma and save time; this ultimately leads to a decrease in the level of discomfort and morbidity that the patient experiences following surgical procedures [55-57]. Flapless implant surgery is a surgical method where the implant osteotomy is prepared and the implant is placed without lifting a mucoperiosteal flap. This surgical procedure is also known as microsurgical implant placement. Flapless implant placement is the preferred procedure when there is a need for quick implant placement in a new extraction socket, in order to maintain the existing vascular supply and soft tissue contouring. This allows for optimal healing of the tissues that surround the implant [58,59].

Techniques for flapless implant placement

**Figure 12:**
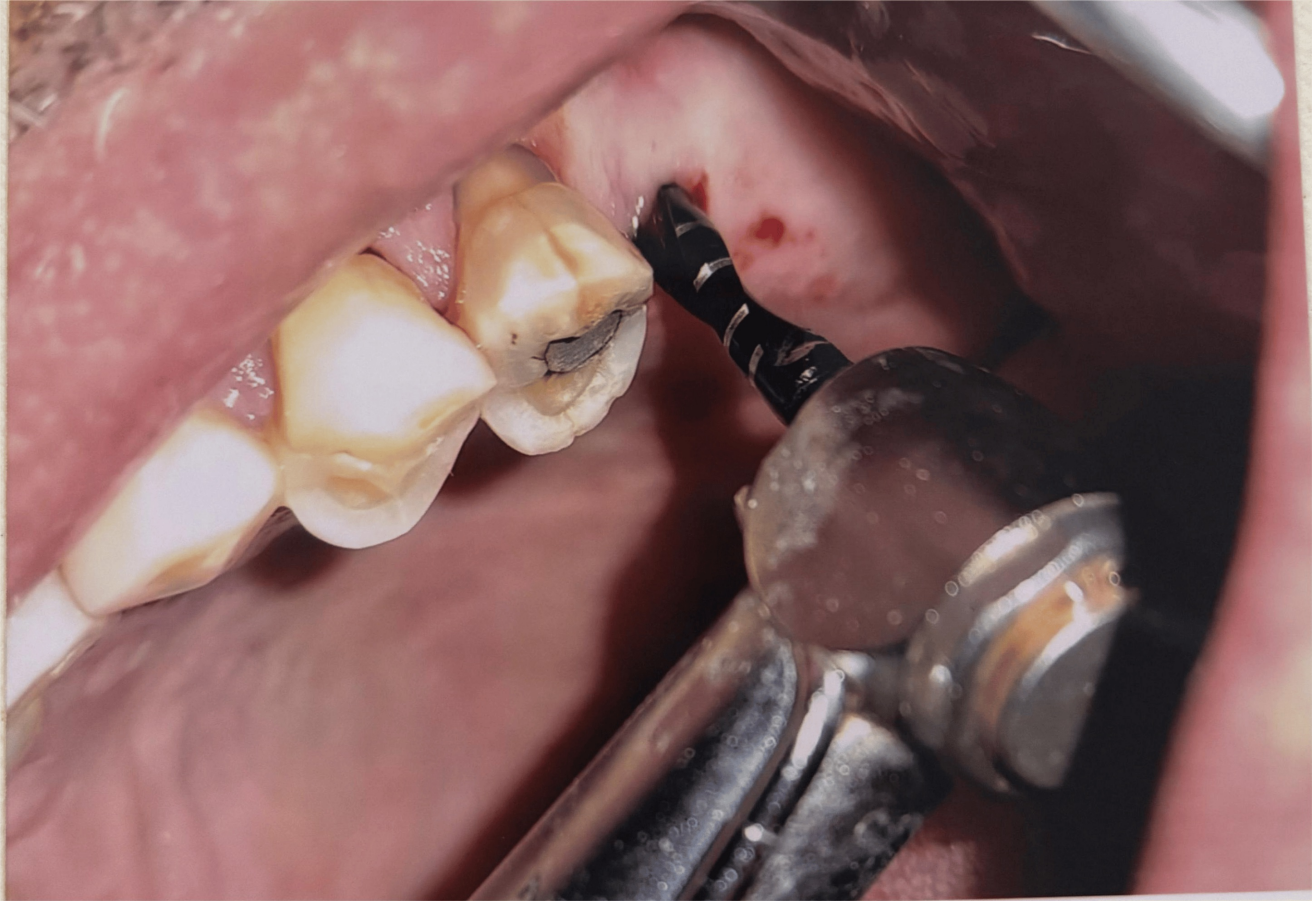
Punch technique Original figure by author

There are several minimally invasive approaches to dental implant placement, each varying in technique and technological integration. One method involves the use of a soft tissue punch, which allows direct access to the drilling site by removing a small circle of gingival tissue at the center of the implant site (Figure 12). Alternatively, a mini incision technique may be employed, wherein a surgical spherical bur is used to transgingivally penetrate both the soft tissue and the underlying bone without requiring flap elevation [60]. Advancing further, guided surgery leverages surgical templates created from maxillary and mandibular impressions, typically processed in a dental laboratory, to ensure precise implant positioning. A more sophisticated approach, guided surgery using 3D navigation, incorporates cone-beam computed tomography (CBCT), advanced implant planning software, and image-guided template generation to facilitate computer-aided surgical procedures. These innovations aim to enhance the accuracy, predictability, and overall success of implant placement while minimizing patient discomfort and recovery time [61].

Innovative recent approaches

**Figure 13:**
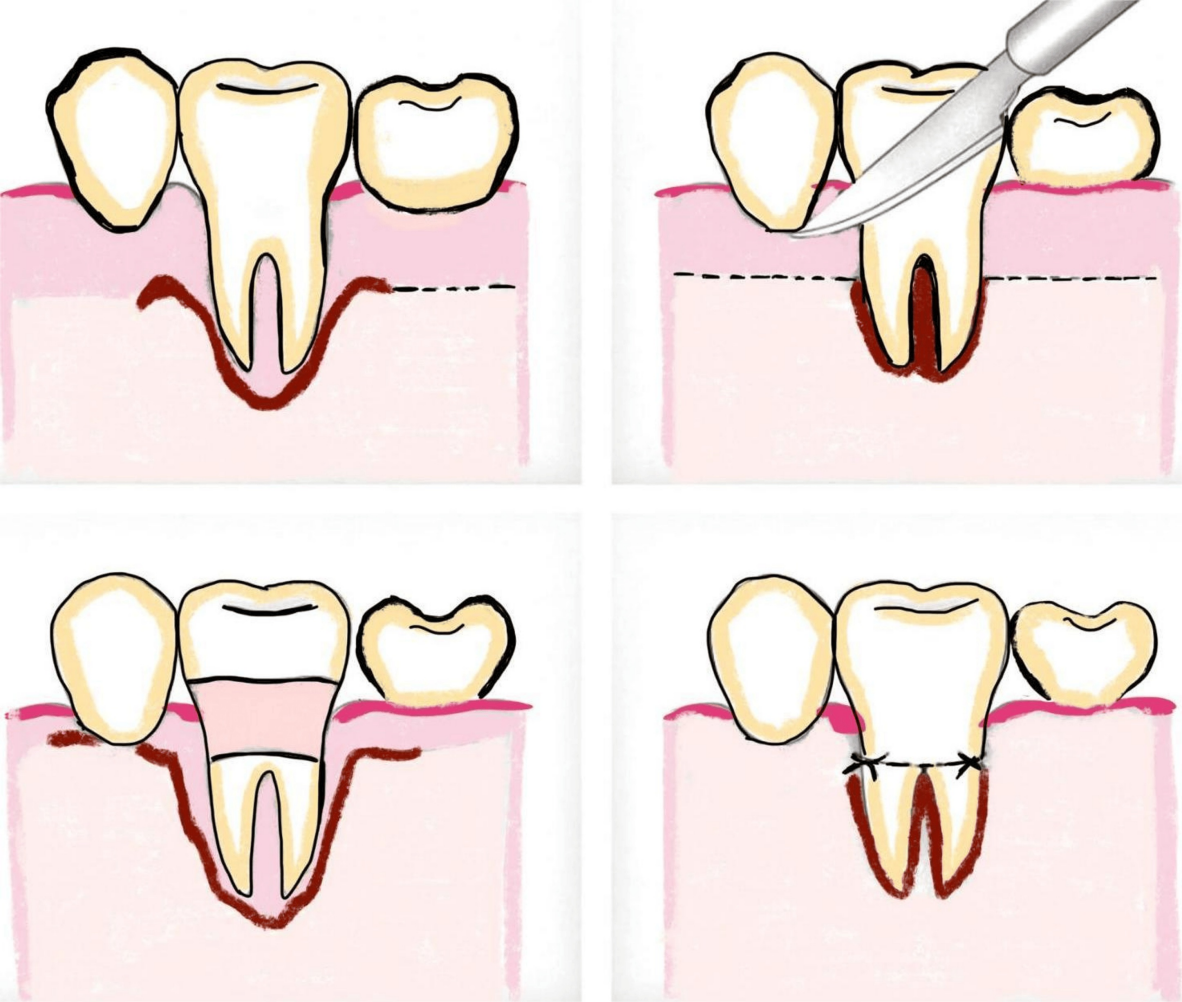
Apically incised coronally advanced surgical technique Original figure by author

The apically incised coronally advanced surgical technique (AICAST) represents a novel surgical method aimed at addressing deep, non-contained intra-bony periodontal defects, while also seeking to minimize related gingival recessions. This procedure entails a horizontal incision in the vestibular mucosa, located a minimum of 10 mm apical to the gingival margin and extending mesiodistally to encompass the affected tooth and its adjacent teeth. A full-thickness mucoperiosteal flap is subsequently elevated coronally towards the gingival margins of the affected teeth (Figure 13). The technique shows significant improvements in clinical attachment levels, effective preservation of marginal soft tissues, and a potential reduction in gingival recession [62].

**Figure 14:**
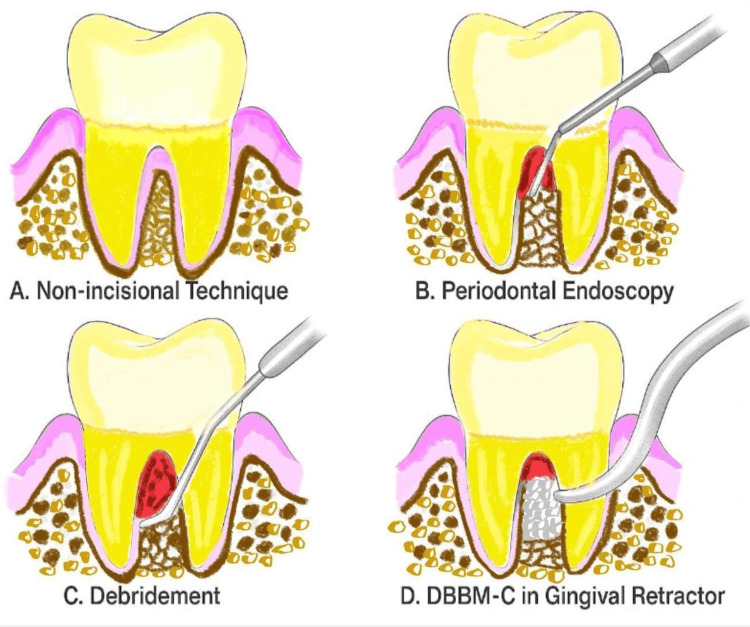
Non-incisional technique Original figure by author

Shi et al. presented a minimally invasive surgical approach termed the non-incisional technique (NIT), which eliminates the need for surgical incisions or flap elevation. Granulation tissue and calculus were excised utilizing real-time magnified endoscopic guidance. Bio-Oss Collagen, a deproteinized bovine bone mineral containing collagen, was meticulously placed into the bone defect with the aid of a gingival retractor, ensuring the preservation of soft tissue integrity and minimizing trauma. The NIT technique, supported by periodontal endoscopy, presents a promising minimally invasive and effective alternative to conventional regenerative approaches for addressing intra-bony defects (Figure 14). The capacity to preserve soft tissue, ensure clot stability, and attain significant clinical attachment improvements renders it a valuable approach in modern periodontal therapy, particularly when aesthetics and patient comfort are priorities [63].

Future potential for minimally invasive periodontal surgery

The medical and dental professions' enthusiastic embrace of minimally invasive surgery as well as nonsurgical therapy has positioned periodontal therapy for future success. Given the progress in technology and the documented benefits of minimally invasive surgical techniques, it is expected that there will be further advancements in this field in the future. Robot-assisted minimally invasive surgery holds the potential to be a groundbreaking advancement in the same direction. Anticipated outcomes of this technique include enhanced precision and skill of the surgeon while minimizing harm to the patient. The utilisation of robotics would enable the execution of surgical procedures to be automated by the surgeon via a remote control.

## Conclusions

Periodontal surgery has undergone significant transformations over the years, transitioning from pocket elimination to pocket reduction and now moving towards the era of regeneration. Surgeons are currently focused on performing surgeries that are more user-friendly, less invasive, and aesthetically pleasing for patients. The idea of "extension for prevention" has been superseded by the concept of "conserve to preserve." 

As a result of the advancement of minimally invasive surgery techniques, there have been numerous benefits, including a shorter duration, improved wound stability due to minimally mobilised flaps, favoured healing, primary closure of the wound that is stable, in addition to a significant decrease in the patient's morbidity both during and following the surgical procedure.

## References

[1] Papapanou PN, Sanz M, Buduneli N (2018). Periodontitis: Consensus report of workgroup 2 of the 2017 World Workshop on the Classification of Periodontal and Peri-Implant Diseases and Conditions. J Periodontol.

[2] Daniel RK (1979). Microsurgery: through the looking glass. N Engl J Med.

[3] Shanelec DA (2003). Periodontal microsurgery. J Esthet Restor Dent.

[4] Jalaluddin Jalaluddin, Mohammad; Agrawal, Urmi; Singh, Dhirendra Kumar (2022). Conceptual approach to periodontal microsurgery: an insight. J Prim Care Dent Oral Health.

[5] Serafin D (1980). Microsurgery: past, present and future. Plast Reconstr Surg.

[6] Barraquer JI (1980). The history of the microscope in ocular surgery. J Microsurg.

[7] Fitzpatrick JM, Wickham JE (1990). Minimally invasive surgery. Br J Surg.

[8] Hunter JG, Sackier JM (1993). Minimally invasive high-tech surgery; into the 21st century. British J Surg.

[9] Tibbetts LS, Shanelec D (1998). Periodontal microsurgery. Dent Clin North Am.

[10] Harrel SK, Ree TD (1995). Granulation tissue removal in routine and minimally invasive procedures. Compend Contin Educ Dent.

[11] Harrel SK, Nunn ME (2001). Longitudinal comparison of the periodontal status of patients with moderate to severe periodontal disease receiving no treatment, non-surgical treatment, and surgical treatment utilizing individual sites for analysis. J Periodontol.

[12] Belcher JM (2001). A perspective on periodontal microsurgery. Int J Periodontics Restorative Dent.

[13] Cortellini P, Tonetti MS, Lang NP (2001). The simplified papilla preservation flap in the regenerative treatment of deep intrabony defects: clinical outcomes and postoperative morbidity. J Periodontol.

[14] Cortellini P, Tonetti MS (2005). Clinical performance of a regenerative strategy for intrabony defects: scientific evidence and clinical experience. J Periodontol.

[15] Cortellini P, Tonetti MS (2007). Minimally invasive surgical technique and enamel matrix derivative in intra-bony defects. I: clinical outcomes and morbidity. J Clin Periodontol.

[16] Cortellini P, Tonetti MS (2009). Improved wound stability with a modified minimally invasive surgical technique in the regenerative treatment of isolated interdental intrabony defects. J Clin Periodontol.

[17] Cortellini P, Prato GP, Tonetti MS (1999). The simplified papilla preservation flap. A novel surgical approach for the management of soft tissues in regenerative procedures. Int J Periodontics Restorative Dent.

[18] Cortellini P, Prato GP, Tonetti MS (1995). The modified papilla preservation technique. A new surgical approach for interproximal regenerative procedures. J Periodontol.

[19] Cortellini P, Pini Prato G, Tonetti MS (1995). Periodontal regeneration of human intrabony defects with titanium reinforced membranes. A controlled clinical trial. J Periodontol.

[20] Pierpaolo Cortellini (2012). Minimally invasive surgical techniques in periodontal regeneration. J Evid Based Dent Pract.

[21] Harrel SK (1999). A minimally invasive surgical approach for periodontal regeneration: surgical technique and observations. J Periodontol.

[22] Sultan N, Jafri Z, Sawai M, Bhardwaj A (2020). Minimally invasive periodontal therapy. J Oral Biol Craniofac Res.

[23] Balakrishnan A, Arunachalam L T, Sudhakar U (2019). Minimally invasive surgery in periodontics - a review. IP Int J Periodontol Implantol.

[24] Trombelli L, Simonelli A, Pramstraller M, Wikesjö UM, Farina R (2010). Single flap approach with and without guided tissue regeneration and a hydroxyapatite biomaterial in the management of intraosseous periodontal defects. J Periodontol.

[25] Aslan S, Buduneli N, Cortellini P (2017). Entire papilla preservation technique in the regenerative treatment of deep intrabony defects: 1-Year results. J Clin Periodontol.

[26] Moreno Rodriguez JA, Caffesse RG (2018). Nonincised papillae surgical approach (NIPSA) in periodontal regeneration: preliminary results of a case series. Int J Periodontics Restorative Dent.

[27] Bianchi AE, Bassetti A (2009). Flap design for guided tissue regeneration surgery in the esthetic zone: the "whale's tail" technique. Int J Periodontics Restorative Dent.

[28] Kuriakose A, Ambooken M, Jacob J, John P (2015). Modified Whale's tail technique for the management of bone-defect in anterior teeth. J Indian Soc Periodontol.

[29] Tarnow DP (1986). Semilunar coronally repositioned flap. J Clin Periodontol.

[30] Allen AL (1994). Use of the supraperiosteal envelope in soft tissue grafting for root coverage. I. Rationale and technique. Int J Periodontics Restorative Dent.

[31] Zabalegui I, Sicilia A, Cambra J, Gil J, Sanz M (1999). Treatment of multiple adjacent gingival recessions with the tunnel subepithelial connective tissue graft: a clinical report. Int J Periodontics Restorative Dent.

[32] Mahn DH (2001). Treatment of gingival recession with a modified "tunnel" technique and an acellular dermal connective tissue allograft. Pract Proced Aesthet Dent.

[33] Zadeh HH (2011). Minimally invasive treatment of maxillary anterior gingival recession defects by vestibular incision subperiosteal tunnel access and platelet-derived growth factor BB. Int J Periodontics Restorative Dent.

[34] Lee CT, Hamalian T, Schulze-Späte U (2015). Minimally invasive treatment of soft tissue deficiency around an implant-supported restoration in the esthetic zone: modified VISTA technique case report. J Oral Implantol.

[35] Zuhr O, Rebele SF, Thalmair T, Fickl S, Hürzeler MB (2009). A modified suture technique for plastic periodontal and implant surgery--the double-crossed suture. Eur J Esthet Dent.

[36] Marques T, Santos NB, Sousa M, Fernandes JC, Fernandes GV (2023). Mixed-thickness tunnel access (Mitt) through a linear vertical mucosal incision for a minimally invasive approach for root coverage procedures in anterior and posterior sites: technical description and case series with 1-year follow-up. Dent J (Basel).

[37] Chao JC (2012). A novel approach to root coverage: the pinhole surgical technique. Int J Periodontics Restorative Dent.

[38] Novaes AB Jr, Grisi DC, Molina GO, Souza SL, Taba M Jr, Grisi MF (2001). Comparative 6-month clinical study of a subepithelial connective tissue graft and acellular dermal matrix graft for the treatment of gingival recession. J Periodontol.

[39] Urban I, Montero E, Sanz-Sánchez I, Palombo D, Monje A, Tommasato G, Chiapasco M (2023). Minimal invasiveness in vertical ridge augmentation. Periodontol 2000.

[40] Sclar AG (2003). The vascularized interpositional periosteal-connective tissue (VIP-CT) flap. Soft Tissue and Esthetic Considerations in Implant Therapy.

[41] Misch CM (2004). Implant site development using ridge splitting techniques. Oral Maxillofac Surg Clin North Am.

[42] Triplett RG, Schow SR (1996). Autologous bone grafts and endosseous implants: complementary techniques. J Oral Maxillofac Surg.

[43] Khoury F, Doliveux R (2018). The bone core technique for the augmentation of limited bony defects: five-year prospective study with a new minimally invasive technique. Int J Periodontics Restorative Dent.

[44] Kao RT, Nares S, Reynolds MA (2015). Periodontal regeneration - intrabony defects: a systematic review from the AAP Regeneration Workshop. J Periodontol.

[45] Aoki A, Mizutani K, Schwarz F (2015). Periodontal and peri-implant wound healing following laser therapy. Periodontol 2000.

[46] Makhlouf M, Dahaba MM, Tuner J, Eissa SA, Harhash TA (2012). Effect of adjunctive low level laser therapy (LLLT) on non-surgical treatment of chronic periodontitis. Photomed Laser Surg.

[47] Qadri T, Poddani P, Javed F, Tunér J, Gustafsson A (2010). A short-term evaluation of Nd:YAG laser as an adjunct to scaling and root planing in the treatment of periodontal inflammation. J Periodontol.

[48] Schär D, Ramseier CA, Eick S, Arweiler NB, Sculean A, Salvi GE (2013). Anti-infective therapy of peri-implantitis with adjunctive local drug delivery or photodynamic therapy: six-month outcomes of a prospective randomized clinical trial. Clin Oral Implants Res.

[49] Kfir E, Kfir V, Eliav E, Kaluski E (2007). Minimally invasive antral membrane balloon elevation: report of 36 procedures. J Periodontol.

[50] Bathla SC, Fry RR, Majumdar K (2018). Maxillary sinus augmentation. J Indian Soc Periodontol.

[51] Kher U, Ioannou AL, Kumar T, Siormpas K, Mitsias ME, Mazor Z, Kotsakis GA (2014). A clinical and radiographic case series of implants placed with the simplified minimally invasive antral membrane elevation technique in the posterior maxilla. J Craniomaxillofac Surg.

[52] Huwais S (2014). WO2014/077920. Geneva, Switzerland: World Intellectual Property Organization Publication. https://patentscope.wipo.int/search/en/detail.jsf?docId=WO2014077920.

[53] Pai UY, Rodrigues SJ, Talreja KS, Mundathaje M (2018). Osseodensification - A novel approach in implant dentistry. J Indian Prosthodont Soc.

[54] Pozzi A, Moy PK (2014). Minimally invasive transcrestal guided sinus lift (TGSL): a clinical prospective proof-of-concept cohort study up to 52 months. Clin Implant Dent Relat Res.

[55] Brodala N (2009). Flapless surgery and its effect on dental implant outcomes. Int J Oral Maxillofac Implants.

[56] Fortin T, Bosson JL, Isidori M, Blanchet E (2006). Effect of flapless surgery on pain experienced in implant placement using an image-guided system. Int J Oral Maxillofac Implants.

[57] Naeini EN, Atashkadeh M, De Bruyn H, D'Haese J (2020). Narrative review regarding the applicability, accuracy, and clinical outcome of flapless implant surgery with or without computer guidance. Clin Implant Dent Relat Res.

[58] Esposito MA, Koukoulopoulou A, Coulthard P, Worthington HV (2006). Interventions for replacing missing teeth: dental implants in fresh extraction sockets (immediate, immediate-delayed and delayed implants). Cochrane Database Syst Rev.

[59] Rocci A, Martignoni M, Gottlow J (2003). Immediate loading of Brånemark System TiUnite and machined-surface implants in the posterior mandible: a randomized open-ended clinical trial. Clin Implant Dent Relat Res.

[60] Jeong SM, Choi BH, Xuan F, Kim HR (2012). Flapless implant surgery using a mini-incision. Clin Implant Dent Relat Res.

[61] Yadav MK, Verma UP, Parikh H, Dixit M (2018). Minimally invasive transgingival implant therapy: a literature review. Natl J Maxillofac Surg.

[62] Calzavara D, Morante S, Sanz J, Noguerol F, Gonzalez J, Romandini M, Sanz M (2021). The apically incised coronally advanced surgical technique (AICAST) for periodontal regeneration in isolated defects: a case series. Quintessence Int.

[63] Shi J, Wang J, Yang Z, Li J, Lei L, Li H (2023). A novel periodontal endoscopy-aided non-incisional periodontal regeneration technique in the treatment of intrabony defects: a retrospective cohort study. BMC Oral Health.

